# Diet in the Driving Seat: Natural Diet-Immunity-Microbiome Interactions in Wild Fish

**DOI:** 10.3389/fimmu.2019.00243

**Published:** 2019-02-19

**Authors:** Ida M. Friberg, Joe D. Taylor, Joseph A. Jackson

**Affiliations:** School of Environment and Life Sciences, University of Salford, Salford, United Kingdom

**Keywords:** diet, teleost, immunity, transcriptome, microbiome, corynebacteria, streptococcus

## Abstract

Natural interactions between the diet, microbiome, and immunity are largely unstudied. Here we employ wild three-spined sticklebacks as a model, combining field observations with complementary experimental manipulations of diet designed to mimic seasonal variation in the wild. We clearly demonstrate that season-specific diets are a powerful causal driver of major systemic immunophenotypic variation. This effect occurred largely independently of the bulk composition of the bacterial microbiome (which was also driven by season and diet) and of host condition, demonstrating neither of these, *per se*, constrain immune allocation in healthy individuals. Nonetheless, through observations in multiple anatomical compartments, differentially exposed to the direct effects of food and immunity, we found evidence of immune-driven control of bacterial community composition in mucus layers. This points to the interactive nature of the host-microbiome relationship, and is the first time, to our knowledge, that this causal chain (diet → immunity → microbiome) has been demonstrated in wild vertebrates. Microbiome effects on immunity were not excluded and, importantly, we identified outgrowth of potentially pathogenic bacteria (especially mycolic-acid producing corynebacteria) as a consequence of the more animal-protein-rich summertime diet. This may provide part of the ultimate explanation (and possibly a proximal cue) for the dramatic immune re-adjustments that we saw in response to diet change.

## Introduction

Interactions between the diet, the microbiome, and the immune system, are increasingly recognized to contribute to health and disease (1–5). Most animal studies addressing these relationships have been carried out in laboratory models or other highly artificial contexts. However, important insights can be gained from natural systems, not least because these reflect the conditions to which host organisms are adapted by natural selection. Any host responses observed in such settings are thus, by definition, more likely to be adaptive—and relatable to an environmental context that may help us understand function.

Here we set out to clarify the driving role of diet in a natural host-symbiont system. We reasoned that large, predictable natural diet changes are particularly likely to provide insights into how the microbiome and immunity are controlled, through generating large, and thus more measurable, responses that are the result of evolutionary adaptation. We focussed specifically on major seasonal diet shifts (6) affecting a wild vertebrate model, the three-spined stickleback (*Gasterosteus aculeatus*) (7, 8).

Importantly, we employed a combination of field observation and complementary experimental manipulation to define the relationship between diet, microbiotal composition and systemic immune expression. In taking this approach we explicitly address the tripartite nature of interactions involving the microbiome. Thus, in the laboratory, the gut microbiota is well-known to undergo a complex two-way cross-talk with the host immune system (9–13) and other physiological responses (14–16). In turn, both are continuously subject to one-way external influence from the diet (17). The latter may directly affect the gut microbiota through availability of substrates and colonizers (though we control the latter here through using partially sterilized foods) (18). Diet may also change the resource status of the host body, leading to functional impairment or strategic re-allocation of immune investments (19, 20) that indirectly modify the microbiota through immune mechanisms.

In our study we initially focussed on wild populations in which we have previously observed a major genome-wide circannual oscillation in the expression of immune-associated, “immunome,” genes (21, 22). (Here and below we refer to “immunome” genes as those with known comparative immune associations (21) in the context of genome-wide analyses.) The above oscillation is partly driven directly by environmental temperature (22, 23) and partly by other, as yet unidentified, seasonal environmental variation that might include variation in diet (18). The oscillation explains more immunome-wide variation than other relevant factors (including geographic site, sex and ontogeny) and is characterized by outlying values in the late winter and late summer. Moreover, immunome genes contributing to the trend comprise two sets tending to fluctuate in antiphase. One set, with maximum expression around late summer, is characterized by genes involved in adaptive effector (lymphocyte) responses, whilst another set, with maximum expression in late winter, lacks such genes and instead contains genes involved in innate immunity and negative regulation of lymphocyte responses.

This remarkable circannual oscillation occurs at the same time as seasonal trends in the stickleback diet (6) and other environmental variables including temperature (22). We thus firstly asked whether these season-specific changes are reflected in the composition of the commensal bacterial community (microbiome). To answer this, we enumerated bacterial reads extracted from RNAseq analyses of RNA pools from the whole bodies of wild fish in winter and summer. Going further, we sought to quantify the causal effects of diet (18) experimentally. For this we carried out a design in which we acclimated wild fish, captured in winter, to outdoor tanks (22, 24). In this approach, we aimed for subjects as phenotypically close to the wild state as possible, whilst maintaining full experimental control (22, 23). Under otherwise common garden winter conditions, these fish were exposed to winter- and summer-specific diets for 3 weeks. We then measured bacterial community composition (by 16S rRNA amplicon sequencing) in gill and intestine and gene expression, for a panel of immune-associated genes, by real-time PCR in multiple tissues.

The above measurements in distinct anatomic compartments were important because they permitted additional inferences about the processes controlling bacterial community composition. Thus, microbes on the gill surface exist entirely associated with mucus layers swept by water currents in which substrates derived from the diet are relatively sparse. On the other hand, the gut contains microbes within mucus layers, but also in large numbers in the lumen (25), where the diet provides a massive source of substrates. This means that any direct (substrate-driven) effect of diet on the microbiome is likely to be important in the gut but much less important on the gill. Equally, the host immune system is likely to be most strongly in contact with microbes in mucus layers (5), so that immune-mediated effects should be relatively more important in the gill than in the gut (where direct diet effects are also important). We therefore predicted that if immune effects are substantial, then the strength of microbiome-immunophenotypic association should be greater in the gill than the gut.

Importantly, the genes whose expression that we measured in our experiment (above) were not arbitrarily chosen but selected to precisely reflect the dominant immunome-wide winter-summer oscillation (21). Also very importantly, we have previously shown the seasonal immune fluctuation to be of great functional relevance, predicting a large component of resistance to an important natural infection (*Saprolegnia parasitica*) when adjusted for thermal effects on parasite establishment (22, 23).

Our study design was thus as strongly cross-referenced to natural variation in the wild as possible and enabled us to quantify diet effects on the microbiome and systemic immunophenotype, going beyond mere correlation. Moreover, through analyses of responses in multiple anatomical compartments (some necessarily more exposed to dietary substrates) we were further able to partition immune-driven effects on the microbiome, from directly diet-driven effects. Taken together, the results presented below give an improved picture of the control and functionality (26) of a natural host-microbiome system, emphasizing the important driving role of diet on immunity.

## Materials and Methods

### Wild Fish: RNAseq for Gene Expression and Bacterial 16S Ribosomal RNA Gene Analysis

Wild fish were sampled from an oligotrophic upland lake (FRN, 52.3599, −3.8776) and lowland river (RHD, 52.4052, −4.0372) in mid Wales in late (astronomical) summer 2012 and late winter 2013 (8/site in summer and 10/site in winter) (A diagrammatic summary of the overall study design is provided in Figure S1). RNA was extracted from whole fish and a genome-wide quantitative gene expression profile generated for each fish by RNAseq, as described in (21). Gene expression data utilized here are expressed in fragments per kilobase of exon per million reads mapped (FPKM), as previously reported. We additionally extracted reads of bacterial 16S rRNA origin from quality controlled Fastq run files for individual fish. Reads were classified against the Greengenes database (operational taxonomic units, OTUs, clustered at 99%) using Taxonomer (27) (for further details, including of controls and identification of possible contaminants, see Supplementary Materials and Methods and Table S1). There were no missing values in the wild fish dataset.

### A Naturalistic Experimental Manipulation of Diet in Mesocosm-Acclimatized Wild Fish

We carried out an experiment simulating season-specific diet at FRN under otherwise common garden conditions (A diagrammatic summary of the overall study design is provided in Figure S1). The seasonal diet treatments were motivated by the finding of Allen and Wootton (6), and our own casual observation (pers. obs.), that during colder periods of the year at FRN, stickleback gut contents can include a substantial proportion of plant-derived detritus and algae. The ingestion of these items may be due to less discriminate benthic foraging during periods of the year when preferred food resources are limited.

**Figure 1:**
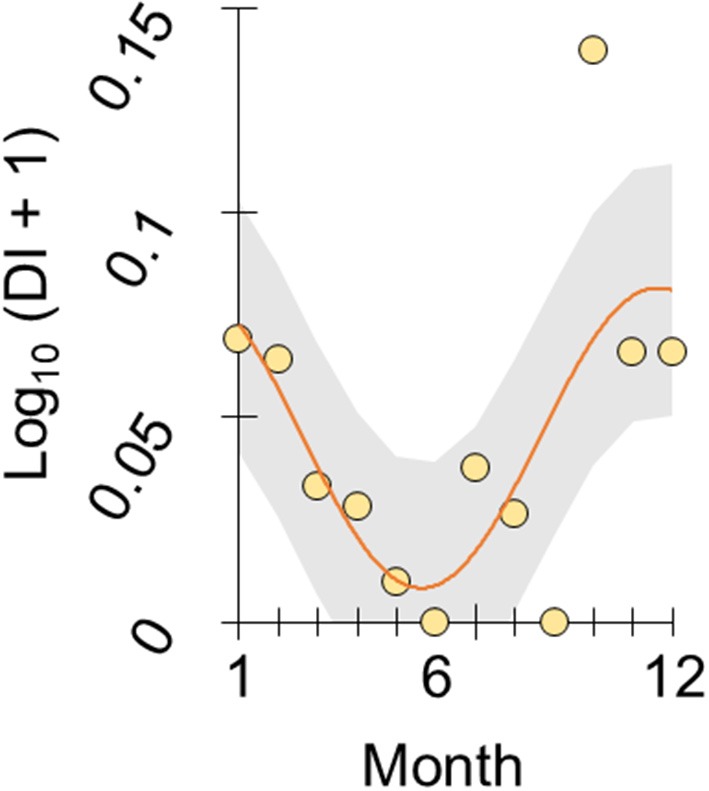
Seasonal diet shift in wild sticklebacks. Plot of log-transformed DI index in stickleback from an upland lake habitat (FRN) against month (1 = January, 12 = December). DI represents the ratio of stomach contents made up by plant material and detritus vs. animals, based on the diet composition data reported by Allen and Wootton (6). The scatter of points represents monthly population-level observations and the line represents a seasonal sinusoid function fitted by cosinor regression with 95% confidence interval shaded.

To illustrate this trend we re-analyzed the year-round monthly stomach content data recorded at FRN by Allen & Wootton (6), according to the following considerations. From Figure 1 in Allen & Wootton (6) we extracted monthly relative diet composition data, using Plot digitizer 2.6.8. An index constructed from these data, of the proportion of stomach contents made up by detritus and plant material as opposed to animals (detritus index, DI), varied seasonally when analyzed by cosinor regression (28) (overall deletion test of sinusoid terms, *p* = 0.05; amplitude parameter, *p* = 0.004; η^2^ = 46.1%, *n* = 12), with lowest predicted values (highest animal content) in June and highest predicted values in January (see Figure 1). Detritus and algae constituted 0–39% of monthly stomach contents.

Taking into account seasonal representation of invertebrates in the study of Allen & Wootton (6) and in other seasonal diet studies of freshwater stickleback in the U.K. (29, 30) we then formulated a summer-like diet treatment consisting of common animal prey items (copepods, cladocerans and chironomid larvae) and a winter-like diet treatment consisting of plant detritus (more typical of stomach contents during winter months) and chironomid larvae (a prey item typically consumed year-round). For the winter-like diet, boiled (1 h), triturated spinach leaves, representing plant detritus, were mixed with triturated gamma irradiated chironomid larvae (Tropical Marine Centre 8153) at a ratio of 3.8 g per 100 ml sieved detritus. The summer-like diet consisted of equal amounts of chironomid larvae, cyclopoid copepods (Tropical marine centre 8171) and cladocerans (Tropical marine centre 8151) (all gamma irradiated). Each diet was provided in excess in each tank and was continuously available *ad libitum*. We confirmed visually (on dissection at the end of the experiment, see below) that fish fed the winter diet ingested substantial amounts of plant detritus during foraging for chironomid fragments.

Fish destined for the experiment (carried out at Aberystwyth University) were captured at FRN in winter (January) and acclimatized to outdoors tanks (exposed to natural photoperiod and temperature) for 1 week, during which they were treated twice with praziquatel, as previously described (24), to remove gyrodactylid and diplostomatid infections. Fish were then exposed to the diet conditions, in the same outdoors tanks (see below), for 21 days. Throughout the experiment, fish were maintained in outdoors 10 L tanks (*n* = 8 fish per tank; 2 tanks per treatment group), exposed to natural photoperiod and temperature variation (4 ± 3°C), in conditioned tap water buffered to a PH of 7.5. Water was changed every day, with food replaced after each water change. Ammonia, nitrite and nitrate levels were monitored daily and remained at negligible levels.

At the end of the experiment fish were individually netted and immediately killed by concussion and decerebration and immersed in RNA stabilization solution. A small longitudinal slit was made in the ventrum of each animal prior to immersion to assist penetration of the stabilization solution to the body cavity. Storage of preserved samples was at −70°C. Weight (mg), body length (snout to tail fork, mm) and sex (based on gonad form and pigmentation) were recorded upon thawing prior to nucleic acids extraction (see below). For each fish, we generated a quantitative gene expression profile by quantitative real-time PCR (QPCR, see below).

### Note on the Interpretation of the Diet Treatment Effects

The anti-microbial treatments applied to the diets (boiling or gamma-irradiation), and also the conduct of the tank experiment under non-sterile conditions, entail that microbes in the foods would not be completely eliminated but would be much reduced and greatly altered in composition. We would thus expect the dietary microbe-driven effect on host immunity, if any, to be very unlike that seen in the wild (where microbial exposures would be very different). Equally, an effect on host immunity through different microbial colonization of the (initially identical) tank waters due to the different foods, if any, would also likely be very different to any exposure in the wild. Moreover, the latter possibility would be limited by regular (24 h) water changes and the low environmental temperature (4 ± 3°C), curtailing microbial growth in the tank water. A similar argument would apply to differential toxicity of the experiment diets (i.e., if these were unrepresentative of the wild diets then different effects on immunity would be expected). As the immunophenotypic responses to diet treatment measured below were identical (rather than different) in form to those occurring in the wild under intentionally similar diet changes, we consider the above possibilities unlikely. We additionally note that a design that totally eliminates notional differential bacterial colonization of the food or environment is only possible in a fully microbe-free system, and that such a system (necessarily including captive-reared hosts) would be so artificial as to offer limited insight in the present case.

### Seasonal Reporter (SR) Genes and QPCR Gene Expression Measurements

For experiment fish we focussed on a set of 5 genes (seasonal reporter, SR, genes) whose expression we have shown to report a major immunome-wide seasonal expression signature (21, 22). These include two genes expressed highly in summer: *cd8a* (Emsembl gene identifier: ENSGACG00000008945) and *foxp3b* (ENSGACG00000012777); and three genes expressed highly in winter: *orai1* (ENSGACG00000011865), *tbk1* (ENSGACG00000000607), and an IL-1 receptor genomic cluster member termed here *il1r*-like (ENSGACG00000001328). For wild fish we extracted expression estimates from RNAseq data and there were no missing values (see above). For experimental fish we measured gene expression in spleen, gill, liver and skin (fin) by QPCR using methods similar to those previously described (22, 24) (see Supplementary Materials and Methods). All 5 genes above were measured in all 4 tissues, with the exception of *orai1*, which was not measured in fin due to a technical omission. QPCR measurements considered below are relative expression values, normalized to two endogenous controls genes and indexed to a common calibrator sample by the ΔΔCt method (see Supplementary Materials and Methods). A limited number of missing values occurred for some tissue-specific expression variables and these are summarized in Table S2.

### 16S Ribosomal RNA Gene Amplicon Sequencing in Experimental Fish

DNA was extracted from intestinal tracts preserved in RNA stabilization solution using the MoBio PowerSoil DNA Isolation Kit and samples (randomized) were passed to a sequencing service (Macrogen) for 16S rRNA sequencing (paired end) on a MiSeq machine (Illumina). DNA quality was assessed by electrophoresis and quantified using Quant-iT™ PicoGreen™ dsDNA Assay Kit by fluorimetry on a Victor^3^ reader (Perkin-Elmer). Libraries targetted the 16S V3 and V4 region (amplicon ~460 bp) with Illumina-recommended primers based on (31) (forward 5′ TCGTCGGCAGCGTCAGATGTGTATAAGAGACAGCCTACGGGNGGCWGCAG; reverse 5′ GTCTCGTGGGCTCGGAGATGTGTATAAGAGACAGGACTACHVGGGTATCTAATCC). Library size distributions were assessed with an Agilent Technologies 2100 Bioanalyzer using a DNA 1000 chip. Following sequencing, Fastq files supplied by the sequencing service were merged using the vsearch script -fastq mergepairs (32), then using usearch v9 (33) quality filtered (expected error < 0.5 removed) and truncated to 440 bp. Sequences were de-replicated, sorted by cluster size and OTUs clustered at 97% using the UPARSE algorithm (34). Suspected chimeric sequences were identified using UCHIME (35) against the fullSilva 97% formatteddatabase (36) and removed. Taxonomy was assigned against the full SILVA 97% OTUs reference database. OTU clusters with <5 reads, or of archaeal, eukaryotic, unassigned or chloroplast origin, were removed from the final OTU table. There were no missing values in this dataset (For further details, including of controls and identification of contaminants, see Supplementary Materials and Methods and Table S3).

### Data Analysis

Analyses were carried out in *R* Version 3.4.4. We employed permutational multivariate analysis of variance using distance matrices (PERMANOVA-DM), implemented in the *vegan* package, to partition variance in bacterial community composition (OTU relative abundance) between host and environmental variables. For consistency, we also used PERMANOVA-DM to partition variation in immunophenotypic measures between the same sets of explanatory variables. Analyses of wild site and experiment datasets were conducted separately. PERMANOVA-DM models of the wild site data reported below analyzed bacterial community composition or immune gene expression variables as the response and contained season (summer/winter), site (river/lake), sex (male/female), and body length (continuous) as explanatory terms. PERMANOVA-DM models of the experiment data reported below analyzed bacterial community composition or immune gene expression variables as the response and contained diet treatment (summer-like/winter-like), sex (male/female), and body length (continuous) as explanatory terms. We additionally considered body condition as an explanatory term in the wild site models, and body condition and tank as explanatory terms in the experiment models, but omitted these from final models as they were always non-significant. Where it was desirable to adjust for the effects of one variable that was colinear with another (diet and condition in the experiment), we limited permutations within strata of the adjusted variable. To consider the association between bacterial community composition and immunophenotypic profile we used Mantel correlations of distance matrices to assess the crude (unadjusted) association and partial Mantel correlations to assess the association adjusted for the effect of season or diet treatment (*vegan* package). Principal co-ordinates analysis (PCO; *labdsv* package) was used to ordinate both the bacterial and immunophenotypic datasets, visualizing the scatter of individuals against the major axes of variation. Manhattan distance was used to construct all distance matrices. Gene expression variables in all of the above analyses were first log transformed (log_10_ *x* or log_10_ [*x* + 1]). *Post-hoc* (to the PERMANOVA-DMs) gene-by-tissue confounder-adjusted analyses of season or diet effects on gene expression were carried out in general linear models (LMs) using the core *lm* command. Prior to these analyses, individual gene expression variables were subject to an optimal power transformation (via a Box-Cox procedure, *MASS* package) and then standardized (zero mean, unit standard deviation) for comparability of parameters.

Consistency of gene expression responses across different tissues (in fish from the experiment) was initially assessed by a pairwise correlation analysis (Pearson coefficients) and by ordination of tissue specific gene expression by PCO (see above). We additionally implemented a multivariate generalized linear mixed model (MGLMM) (37, 38) of multiple gene expression responses to diet in different tissues, with individual identity as a random term (*MCMCglmm* package). This allowed estimates of repeatability (across tissues) for individual genes and the estimation of a diet × tissue interaction term to assess the existence of a consistent (systemic) response (i.e., where interaction is important, this is evidence for a lack of consistency across tissues). Gene expression data were log-transformed and standardized for this analysis.

Comparing our two datasets (wild fish at different seasons and experimental fish with different season-specific diet) we also asked whether there was any consistency in the bacterial OTUs associating with season and diet treatment. In each datasest, we ran individual confounder adjusted permutation tests (based on LMs; *lmPerm* package) for each OTU (relative abundance) against season or season-specific diet treatment, identifying genes that were significant at a false discovery rate (FDR) cut-off of *P* = 0.05. We additionally ran random forests machine learning analyses (39) to predict season or diet treatment from the bacterial datasets, identifying variables with high importance.

Whilst our analyses of bacterial variation concentrated on community composition, we did secondarily consider rarefied species richness (in LMs with explanatory terms equivalent to those in the PERMANOVA-DMs used to analyse community composition). The only significant trends were modest associations with body length, which might possibly be due to the dynamics of colonization in wild fish.

Where body condition measures are considered below we analyzed both the residuals from a quadratic regression of body weight on length (RBW) (40) and the scaled mass index (SMI) (41) (SMI and RBW being highly correlated). Term-specific effect size measures presented below are partial *R*^2^ for PERMANOVA-DM and classical η^2^ for LMs.

### Data Availability

The basic data from this study will be available in the European Nucleotide Archive (primary accession number PRJEB13319).

### Note on Comparison of RNAseq and 16S Amplicon Bacterial Composition

We note that inherently different nucleic acid extraction, sequencing and bioinformatics methodologies in our RNAseq and 16S amplicon datasets, likely leading to different biases, make inference from differences between these untenable (i.e., methodology is confounded with dataset). However, we *a priori* determined a strategy of only searching for, and deriving inference from, commonalities between the datasets. Such commonalities, emerging despite methodological differences, are likely to be more robust even than the case of a common methodology (where shared methodological biases might lead to systematic error).

## Results

### Bacterial Community Composition and Immune Allocation Showed Seasonal Responses, but Were Largely Independent of Each Other, in Wild Fish

**Figure 2:**
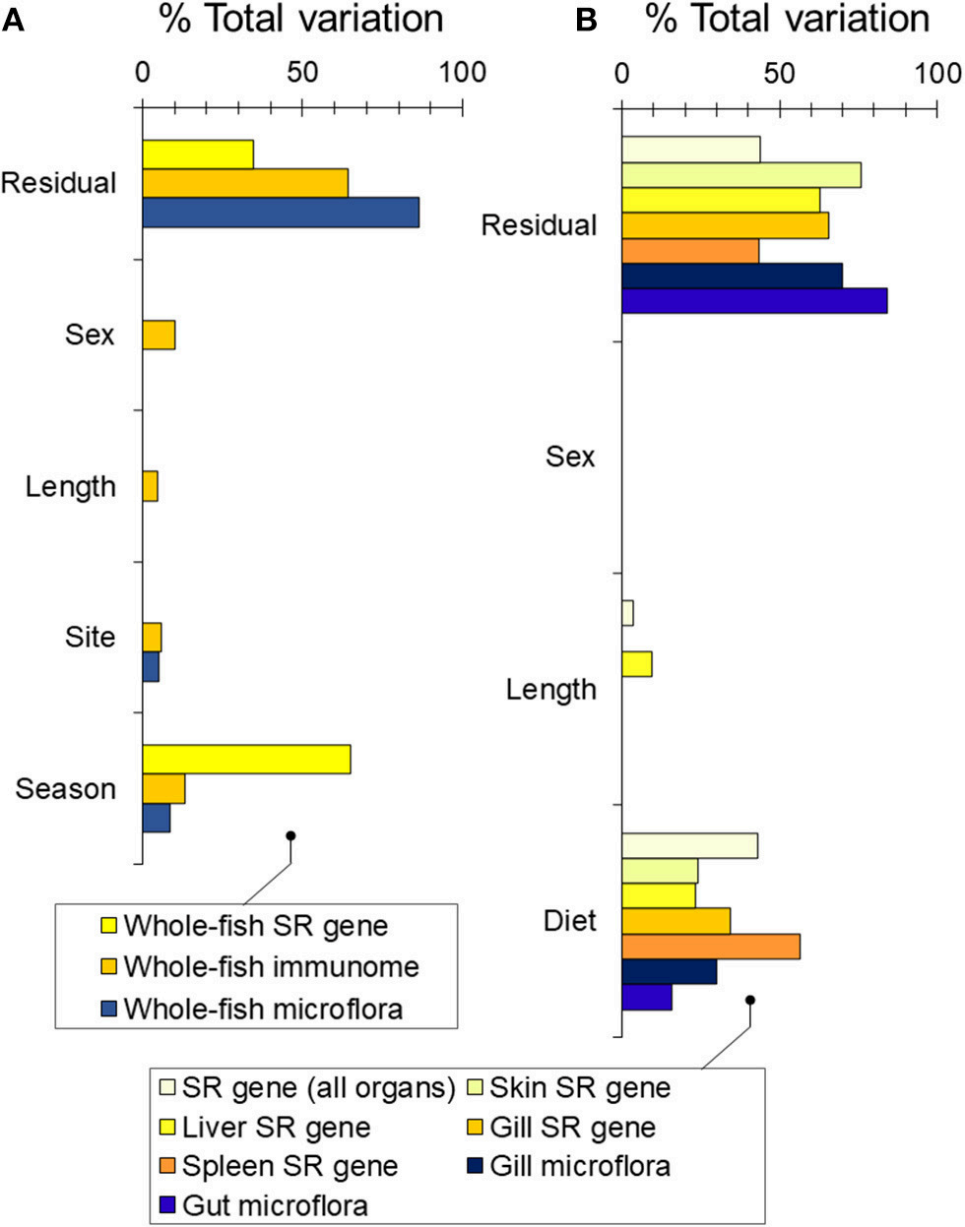
Sources of variability (expressed as partial *R*^2^) in bacterial community composition and gene expression partitioned by PERMANOVA-DM. **(A)** Based on RNAseq analysis of wild fish sampled in winter and summer at FRN. **(B)** Based on bacterial 16S rRNA amplicon sequencing and QPCR gene expression analysis of experimental fish exposed to season-specific diets.

We first asked whether wild sticklebacks differed in winter and summer. We found a modest effect of season on bacterial community composition [RNAseq data; PERMANOVA-DM, *F*_(1, 33)_ = 3.32, *P* < 0.001, *R*^2^ = 8.7%], a weak effect of site [*F*_(1, 33)_ = 1.91, *P* = 0.012, *R*^2^ = 5.0%] and no effect of sex or body length (Figure 2A). Season was a substantial source of variation in immunome-wide [RNAseq data; *n* = 3,648 genes; PERMANOVA-DM, *F*_(1, 31)_ = 6.29, *P* < 0.001, *R*^2^ = 13.1%] and SR gene expression [RNAseq data; *n* = 5 selected seasonal immunome genes; *F*_(1, 34)_ = 63.79, *P* < 0.001, *R*^2^ = 65.2%] (Figure 2A; see also Figure S2), as previously reported (21). Amongst the SR genes, *orai1, tbk1*, and *il1r*-like were expressed relatively more in winter (winter-biased) and *cd8a* and *foxp3b* were expressed more in summer (summer-biased), also as previously reported (21). Bacterial community composition was uncorrelated with immunome-wide expression but significantly correlated with SR gene expression (Mantel *r* = 0.21, *P* = 0.001), though this association disappeared when season was adjusted for. Body condition (RBW and SMI) did not vary between the winter and summer fish [see also (24)] and had no association with bacterial community composition or immune gene expression when added to the PERMANOVA-DM models above.

### An Experiment Simulating Natural Season-Specific Diet Shift Under Otherwise Common Garden Conditions Re-capitulated Seasonal Immune Allocation Changes in the Wild

To assess the causal effect of diet in driving the seasonal changes seen in the wild, we carried out an experiment in which we provided season-specific diets under otherwise common garden conditions. For this experiment, we acclimatized wild fish (captured in January) to outdoors aquaria and applied the diet treatments under ambient temperature and photoperiod conditions. For representativeness, we monitored gene expression in spleen, liver, gill, and fin.

**Figure 3:**
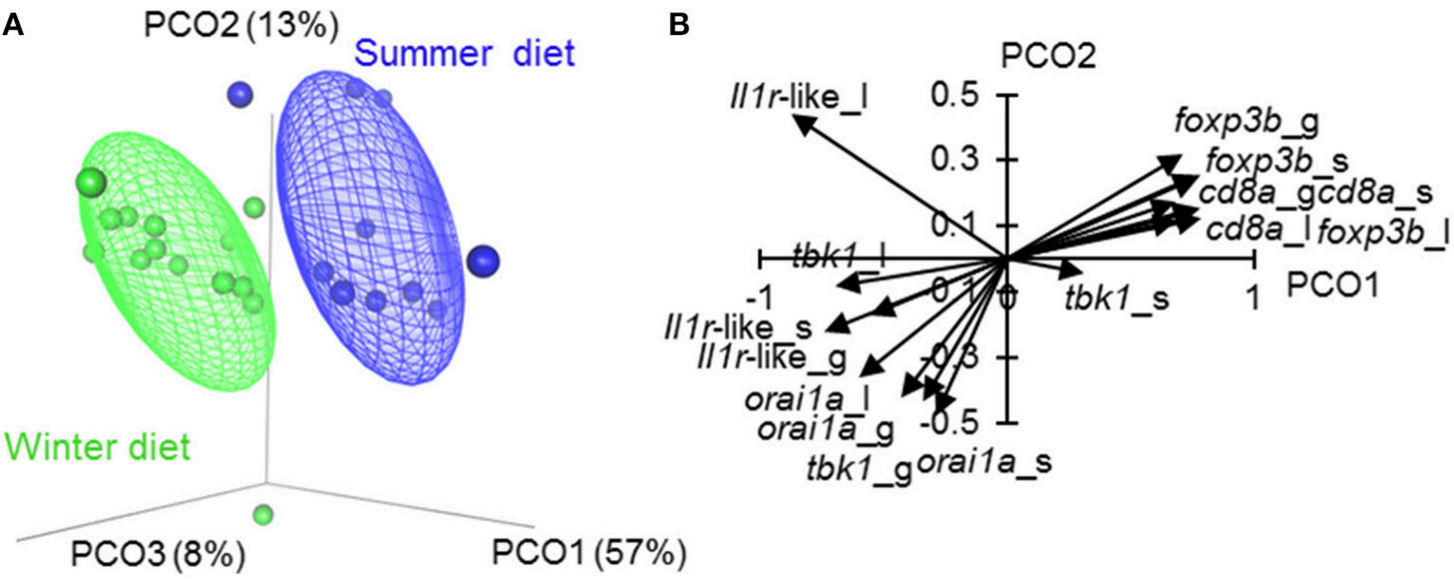
Ordination of tissue-specific immune-associated (SR) gene expression in fish fed season-specific diets under otherwise common garden conditions. **(A)** Individual scatter plotted against the three major axes from a principal coordinates analysis (PCO); percentage of total variation explained indicated for each axis; ellipsoids enclose 50% of distribution. **(B)** Biplot-like plot for the largest two major axes of the SR gene PCO, indicating correlations of tissue-specific gene expression variables with the principal coordinate scores (i.e., positively correlated variables tend to have similar vectors in the plot, and negatively correlated variables tend to have vectors in opposed directions; longer vectors reflect stronger correlations). Plotted vectors are for spleen (_s), liver (_l) and gill (_l). The PCO **(A, B)** omits fin measurements to allow assessment of *orai1* (which was not measured in fin, see Materials and methods).

**Figure 4:**
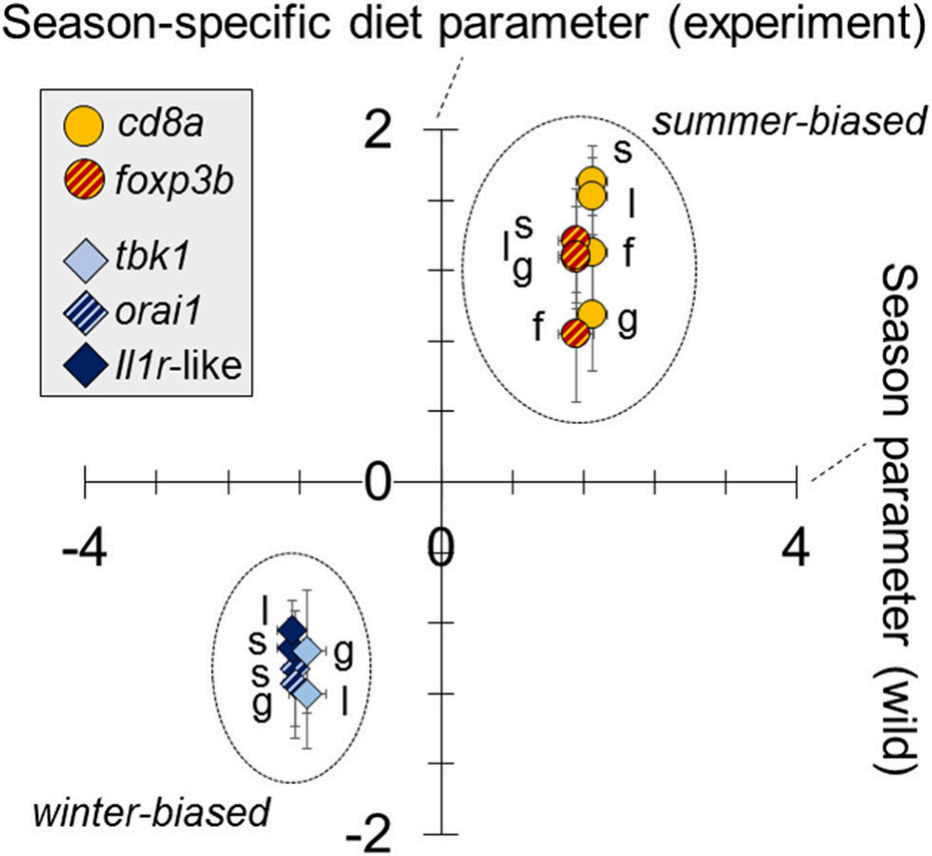
Similar responses to season (in wild fish) as to season-specific diet (in experimental fish) in the expression of immune-associated (SR) genes. Tissue-specific diet parameters for experimental fish (y—axis) (analysis based on QPCR data) are plotted against whole-fish season parameters for wild fish (x—axis) (analysis based on RNAseq data). Parameters are from general linear models (LMs) (one model per sample-specific expression variable per dataset [experiment or wild]), adjusted for the effects of sex and body length (and site, in the case of wild fish). All gene expression variables were standardized (zero mean, unit standard deviation), following Box-Cox transformation, for comparability of the parameters; the parameter reference level is winter for wild fish and winter-specific diet for experimental fish. For each gene there is a single (whole fish) datum for wild fish and up to 4 tissue-specific data for experimental fish (spleen, s; gill, g; liver, l; fin, f). Only significant parameters are plotted.

We found a dominant overall effect of diet on SR gene expression [QPCR data; PERMANOVA-DM, *F*_(1, 15)_ = 11.80, *P* < 0.001, *R*^2^ = 44.8%] (Figures 2B, 3A,B; see also Figure S3) and diet was also the dominant source of variation in all individual tissues (compared to sex and body size) (Figure 2B). For all tissues the summer-like diet forced SR gene expression changes in the same direction as winter to summer changes in the wild (Figure 4). In *post-hoc* analysis of individual genes, those that have previously been established to be winter-biased in the wild (*orai1, tbk1*, and *il1r*-like) [see (21, 22)] tended to be down-regulated, whilst known summer-biased genes (*cd8a* and *foxp3b*) tended to be up-regulated, by the summer-like diet (Table 1). Stronger and more consistent diet effects were observed for T-cell-associated genes (especially *cd8a* and *foxp3b*), but significant trends, in the expected season-specific direction, were observed for all SR genes in one or more organs. There were no effects of sex or body length on immune expression (and no sex by diet interaction) (PERMANOVA-DM) and no effects of body condition (RBW and SMI; accounting for diet effects in a stratified PERMANOVA-DM).

**Table 1:** Diet effects on organ-specific expression of immune-associated genes known to be seasonally expressed in the field (seasonal reporter, SR, genes).

**Gene**	**Bias**	**Tissue**	**Parameter**	**Test statistic**	***P***	**η^**2**^%**
*cd8a*	S	Spleen	−1.71 ± 0.20	*F*_(1, 24)_ = 70.91	1.26 × 10^−8^	71.1
		Gill	−0.95 ± 0.32	*F*_(1, 26)_ = 8.87	6.2 × 10^−3^	23.7
		Liver	−1.62 ± 0.22	*F*_(1, 26)_ = 53.45	9.18 × 10^−8^	66.5
		Fin	−1.30 ± 0.31	*F*_(1, 24)_ = 17.27	3.6 × 10^−4^	41.5
*foxp3b*	S	Spleen	−1.37 ± 0.29	*F*_(1, 24)_ = 21.48	1.1 × 10^−4^	47.2
		Gill	−1.27 ± 0.29	*F*_(1, 26)_ = 18.92	1.9 × 10^−4^	41.0
		Liver	−1.29 ± 0.27	*F*_(1, 26)_ = 23.50	5.0 × 10^−5^	44.4
		Fin	−0.84 ± 0.39	*F*_(1, 22)_ = 4.79	0.039	17.6
*orai1*	W	Spleen	1.06 ± 0.33	*F*_(1, 24)_ = 10.48	3.5 × 10^−3^	27.2
		Gill	1.14 ± 0.31	*F*_(1, 26)_ = 13.03	1.3 × 10^−3^	32.3
		Liver			ns	
*il1r*-like	W	Spleen	0.94 ± 0.33	*F*_(1, 24)_ = 7.93	9.6 × 10^−3^	22.9
		Gill			ns	
		Liver	0.84 ± 0.29	*F*_(1, 26)_ = 8.48	7.3 × 10^−3^	19.3
		Fin			ns	
*tbk1*	W	Spleen			ns	
		Gill	0.96 ± 0.35	*F*_(1, 26)_ = 7.67	0.010	22.6
		Liver	1.20 ± 0.31	*F*_(1, 26)_ = 14.49	7.7 × 10^−4^	35.6
		Fin			ns	

*Results from general linear models (LMs) with tissue-specific QPCR gene expression measurements as the response and diet treatment, sex and body length as explanatory terms. Table shows the parameter estimates ± standard errors, test statistics, P-values and classical eta squared effect sizes for diet. Negative parameters indicate higher expression in fish fed a summer-like diet. Also indicated is the expression bias typically observed in the wild (21, 22): S, summer-biased (high expression in summer); W, winter-biased. Individual gene expression variables were subject to an optimal power transformation (via a Box-Cox procedure) and then standardized (zero mean, unit standard deviation) for comparability of parameters. Parameter estimates above thus correspond to between-group mean differences in these transformed and standardized variables. Of 19 organ × gene combinations, 14 (74%) showed a significant diet effect, in many cases with a very substantial effect size; in all significant cases (14/14, 100%) the direction of the season-specific diet effect corresponded to the expected seasonal bias (see also Figure S3)*.

### An Individually Consistent Immunophenotypic Response to Experimental Diet Treatment Occurred Across Tissues

**Figure 5:**
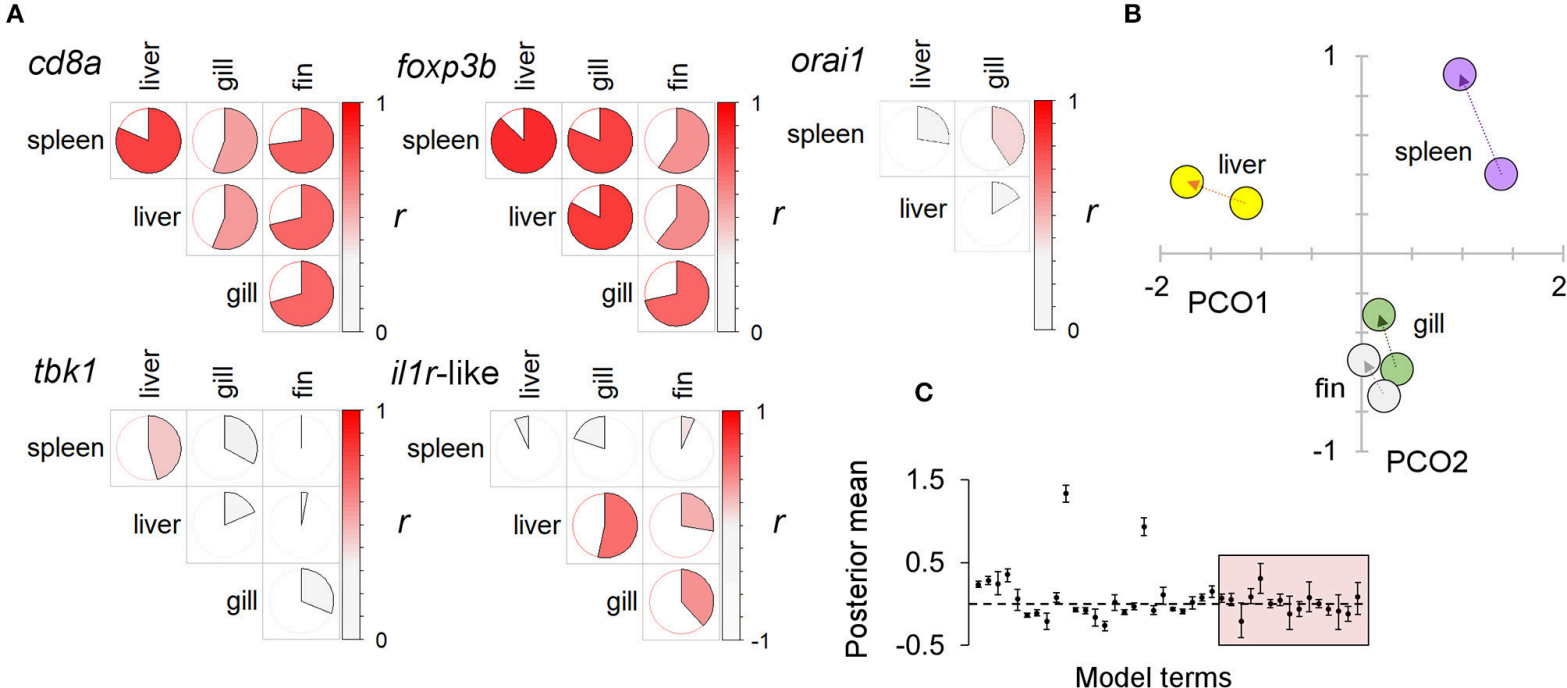
A systemic immunophenotypic response to diet treatment: consistent responses in multiple tissue types. **(A)** Pairwise correlations between the expression of individual immune-associated (SR) genes in different tissues. Circle charts indicate the Pearson correlation coefficient of log-transformed data (full circle, *r* = 1); the strength and direction of the correlation is also indicated by a color scale (to the right in each panel). Higher levels of correlation in T-cell associated genes (*cd8a, foxp3b*) suggest these may disproportionately contribute to consistent responses between tissues. Note: *orai1* was not measured in fin (see Materials and methods). **(B)** Ordination of immune associated (SR) gene expression in different tissues under different diets (against the two major axes from a principal coordinates analysis, PCO). For each tissue an arrow extends between the centroids of the winter and summer diet groups (winter centroid proximal). The similar trajectory of responses in different tissues is suggestive of a consistent systemic response to diet. **(C)** Results from a multivariate generalized linear mixed model (MGLMM) of multiple gene expression responses to diet in different tissues, with individual identity as a random term. Plot of gene-specific posterior mean parameters; 95% highest posterior density intervals indicated by bars. Box encloses diet × tissue parameters; although sometimes significant these are generally less important than other effects, supporting a broadly consistent (systemic) response to diet across tissues.

There was a substantial element of individual consistency in the gene expression responses across different tissues, especially for those genes showing strong responses. Thus, T-cell associated genes (*cd8a, foxp3, orai1*) had large across-tissue repeatabilities (0.4–0.5), but other genes showed non-significant repeatability overall. Nonetheless, tissue-specific responses of all individual genes tended to be positively correlated amongst different tissues and to respond to diet treatment along a consistent multivariate trajectory (in a MGLMM, tissue × treatment interaction was significant overall, *p* = 0.005, but not a comparatively large effect) (Figure 5). Taken together, there was thus strong evidence of a prominent systemic trend in the immunophenotypic response to diet.

### Experimental Diet Treatment Drove Changes in Body Condition Not Seen in the Wild

**Figure 6:**
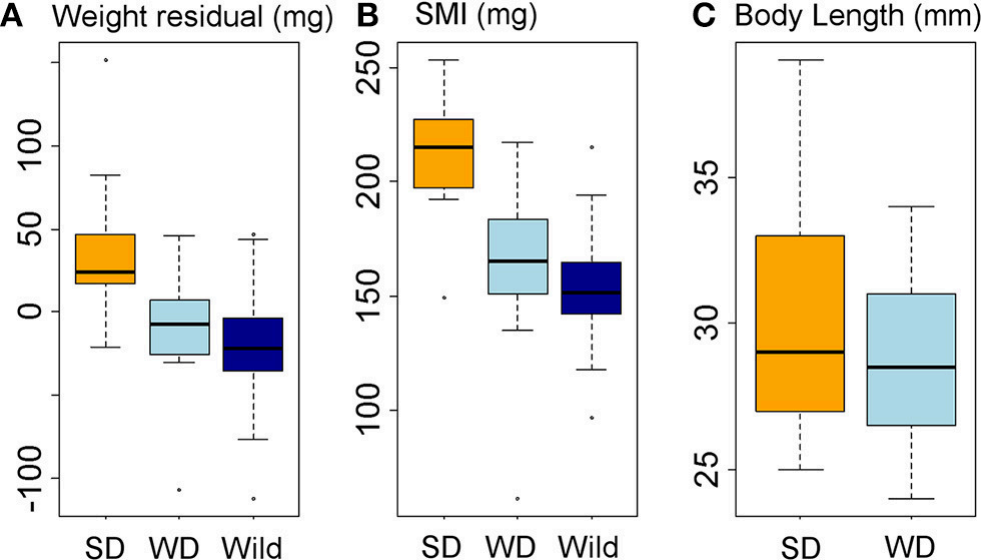
Body condition and body size responses to diet treatment. Data represented in box-and-whisker plots showing median (middle line), second and third quartiles (box), range (whiskers), and outliers (points). **(A, B)** Body condition in diet treatment groups and wild fish, expressed as residual body weight, RBW **(A)** and scaled mass index, SMI **(B)**. **(C)** Body length in diet treatment groups. X-axis labels: SD, summer-like diet treatment; WD, winter-like diet treatment; wild, wild fish sampled in January-February.

The winter-like diet maintained fish in similar body condition to wild fish in January and February (Figures 6A,B). In great contrast to the lack of winter-summer variation in condition seen in wild fish (see above), the summer-like diet promoted a substantial increase in body condition compared to both the winter-like diet (RBW, LM parameter = 48.0 ± 13.2 mg, tukey *P* = 0.002; SMI, LM parameter = 48.3 ± 10.3 mg, tukey *P* < 0.0005) and wild winter fish (RBW, LM parameter = 57.4 ± 12.9 mg, tukey *P* < 0.0005; SMI, LM parameter = 60.0 ± 10.0 mg, tukey *P* < 0.0005) (Figures 6A,B). There was a slight, but non-significant, increase in mean length in the summer-like diet group, compared to the winter-like diet group (which were size matched at the start of the experiment) (Figure 6C).

### Bacterial Community Composition Was Driven by Diet Shift in Experimental Fish and the Gill Community Was More Strongly Associated With Systemic Immune Expression Than the Gut Community

**Figure 7:**
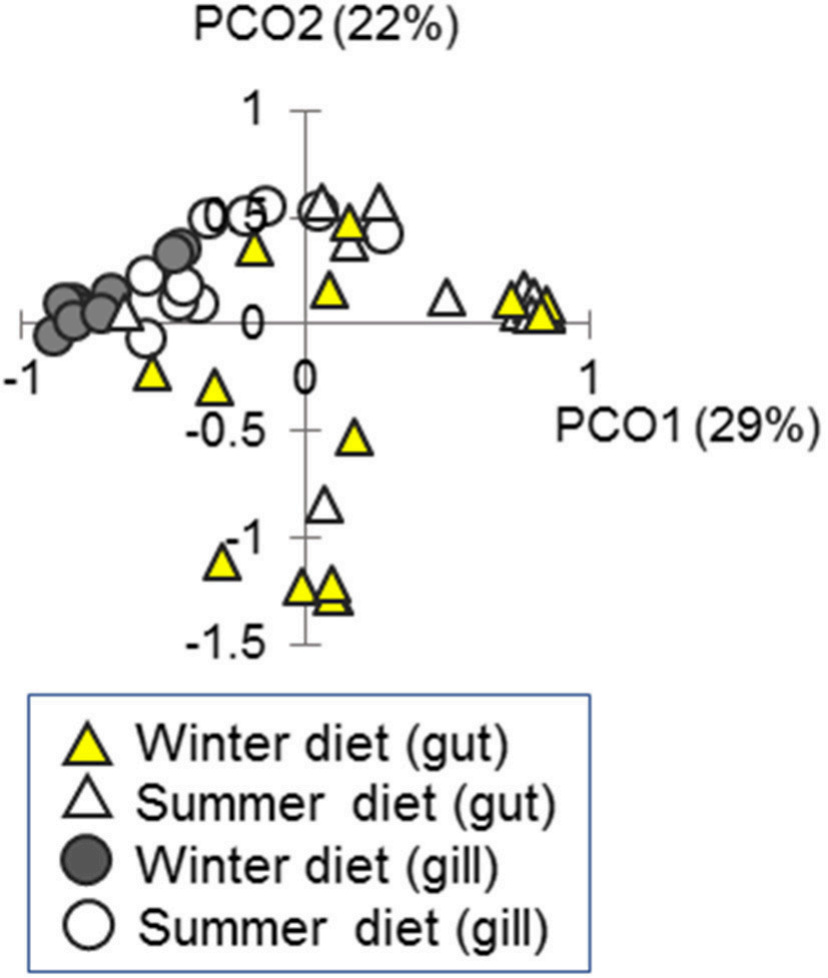
Ordination of bacterial community composition in the gut and gill in fish fed season-specific diets under otherwise common garden conditions. Individual scatter plotted against the two major axes from a principal coordinates analysis (PCO); percentage of total variation explained indicated for each axis.

We measured bacterial community composition in the gut and gill in the above experiment (Figures 2B, 7). There was a substantial effect of diet on bacterial community composition in the gut [16S amplicon sequencing data; PERMANOVA-DM, *F*_(1, 25)_ = 4.66, *P* = 0.002, *R*^2^ = 15.7%] and gill [*F*_(1, 16)_ = 6.87, *P* < 0.001, *R*^2^ = 30.0%], but no effects of sex or body length (and no sex by diet interaction). Body condition (RBW and SMI) was not associated with bacterial community composition in gut or gill if the effect of diet treatment was accounted for (stratified PERMANOVA-DM). We also tested our prediction (above) that the correlation of microbiome composition with immune expression would be stronger in the gill than the gut. This prediction was confirmed when local microbiome composition was compared, in a planned comparison (unadjusted for diet effect), to systemic (splenic) SR gene expression: Mantel *r* for the gill was 0.64 (*p* = 0.001, 95% CI = 0.51–0.72, *n* = 28) and only 0.17 (*p* = 0.014, 95% CI = 0.09–0.27, *n* = 28) for the gut (Splenic expression was chosen for the above comparison because spleen was considered likeliest to reflect systemic responses and also showed the largest diet effect size). Adjustment for diet effect tended to negate the association between the microbiome and splenic SR gene expression, which became non-significant for the gut microbiome and only marginally significant for the gill microbiome.

### Mycolic Acid-Producing Corynebacteria Increased Under Summer-Like Conditions

**Figure 8:**
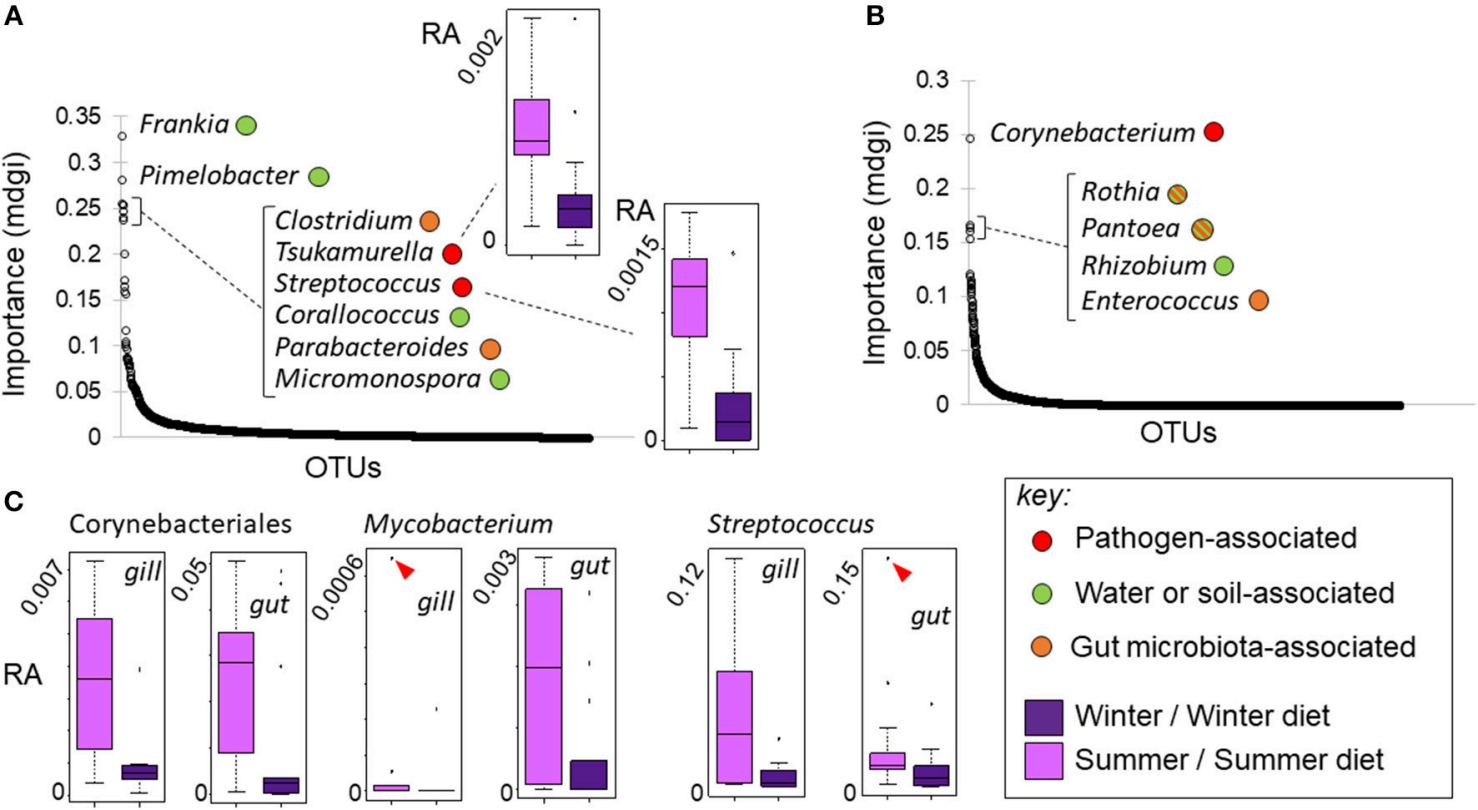
Changes in bacterial OTU relative abundance in response to season in wild fish and season-specific diet in experimental fish. **(A, B)** OTUs ranked by importance (mdgi, mean decrease in Gini index) in random forest analyses discriminating season in wild fish **(A)** and diet in experimental fish **(B)**; typical ecological relationships of the most important genera are indicated (see key). Mycolic acid-producing Corynebacteriales OTUs were predictive of both season in wild fish (*Tsukamurella* sp.) and season-specific diet (*Corynebacterium* sp.) in experimental fish, increasing in the summer condition in both cases. Box-and-whisker plots in insets show relative abundance (RA) responses of selected taxa. **(C)** Box-and-whisker plots showing tissue-specific relative abundance responses of potentially pathogenic taxa in experimental fish. Corynebacteriales, *Mycobacterium* and *Streptococcus* all increased significantly (as groups) in summer diet fish. Markers indicate extreme outlying observations; differences were still significant when these were removed.

There was no genus-level consistency in the bacterial lineages that best predicted season in the wild, compared to the lineages that best predicted season-specific diet treatment in our experiment (Figure 8). Strikingly, however, some of the OTUs most predictive of season and diet treatment were mycolic acid-producing Corynebacteriales that increased in abundance under summer conditions. More specifically, a *Tsukamurella* lineage was 4th most predictive of season out of 1,816 lineages (individually significant with FDR adjustment) (Figure 8A). At the same time a *Corynebacterium* lineage was by far the most predictive of diet treatment out of 3,398 gut lineages (Figure 8B), and the only lineage in which the diet effect was significant after FDR error rate adjustment. *Post hoc* analyses indicated that Corynebacteriales as a whole, and the frequently pathogenic corynebacterial genus *Mycobacterium* individually, increased significantly (*p* < 0.05) in relative abundance in the gill and gut of experimental fish fed the summer-specific diet (Figure 8C). Although the corresponding trends were not observed in wild fish, the relative abundance of a further corynebacterial genus, *Gordonia*, did increase significantly in summer. We note that, whilst less frequently pathogenic than *Mycobacterium, Corynebacterium, Tsukamurella*, and *Gordonia* have all sometimes been associated with disease in fish (42–44). We also note that a lineage of the potentially pathogenic firmicute genus *Streptococcus* was the 5th most predictive of the summer condition in wild fish (Figure 8A), increasing in relative abundance. *Post hoc* analysis revealed this genus also increased in both the gut and gill of experimental fish fed a summer-like diet (*P* < 0.05; Figure 8C).

## Discussion

Using a combination of field observation and experiment in wild fish, we have identified a powerful role for diet in driving systemic immune variation and have provided the first evidence for diet-driven immune control of the microbiome in a natural system. Furthermore, we document the outgrowth of potentially pathogenic bacteria (45) as a consequence (rather than just a correlate) of diet change. This implicates such outgrowths as part of the selective landscape in which diet-driven systemic immune re-adjustments may have evolved.

In our study, we first identified a pattern of seasonal variability in bacterial community composition. When variation in bacterial community composition in wild fish was partitioned amongst season, site and host variables, season greatly dominated, although its effect size was modest in relation to the residual (inter-individual) variation. Seasonal effects also, as we have previously reported (21, 22), were a major source of immunophenotypic variation in the wild fish.

We then attempted to re-create the immunophenotypic and microbiome changes seen in the wild through an experiment, specifically asking whether these might be driven by season-specific diet. In this experiment, wild fish were acclimated to outdoors tanks (in winter) and then subject to common garden conditions, except for a manipulation of diet that mimicked previously observed season-specific diet change (6) at the study site.

We found that provision of a season-specific diet recapitulated the seasonal change in systemic immune-associated gene expression seen in wild fish (with consistent expression responses in different tissues). Diet may therefore contribute to the seasonal immunophenotypic trends in the wild that we have previously demonstrated to be partly driven by temperature (22, 23). Starting from a winter state, fish (captured in the wild during winter) fed a summer-like diet re-adjusted their gene expression toward the state typical of summer in the wild, even if still experiencing otherwise winter conditions. On the other hand, control fish fed a winter-like diet (simulating that at the time of capture) continued to express an unchanged winter-like phenotype. The diet treatment also drove a change in bacterial community composition similar in magnitude to the seasonal change seen in the field. Whilst there was no consistency in the genus identity of OTUs that, respectively best characterized these changes, there was some pattern in the their biological characteristics, which we discuss further below.

Our experiment demonstrated clearly that body condition can be driven into a crude circumstantial correlation with immunophenotypic variation by diet manipulation. Nonetheless, considered as a whole, our results also demonstrate that body condition does not necessarily constrain even major immunophenotypic change in healthy fish. Thus, fish fed a winter-like diet in the experiment maintained the same body condition (weight residual on length) as wild fish in winter. On the other hand, fish fed a summer-like diet strongly upwardly re-adjusted their body condition (coinciding with the immunophenotypic change described above). In contrast, such a re-adjustment of body condition does not occur seasonally in wild fish. Possibly this is because, during summer, energy is directed toward other life history components, rather than to increasing condition. The very similar immunophenotypic re-adjustments (see above) seen in both wild and diet-manipulated fish therefore occur independently of very different body condition responses. This observation corresponds to our further observation that body condition was largely independent of immunophenotypic variation within both the experimental and field sample sets, when season or diet were accounted for. Taken together, these considerations support that diet-driven immunophenotypic re-adjustments may be decoupled from condition or resource “richness” *per se* and could, rather, be a response to percieved disease risk in the environment.

When we focussed on the wider biological context of bacterial OTUs, we did, in fact, find evidence that the microbiome presents a changing disease risk, perceivable by the host. Thus, the changes most predictive of summer in the wild, and of a summer-like diet in the laboratory, involved an increase in potentially pathogenic mycolic acid-producing Corynebacteriales lineages. This is notable because mycolic acids, synthesized exclusively by certain corynebacterial genera (46), are well-known to be important conserved pathogen-associated molecular patterns (PAMPs), modulating the immune system in mammals (47–49).

Changes in Corynebacteriales were more marked in the feeding experiment. This may be due to the short-term nature of the diet treatments causing a temporary imbalance that would be accommodated over longer time periods. Amongst the mycolic acid-producing corynebacterial lineages showing winter-summer or diet-related changes, *Tsukamurella, Gordonia, Corynebacterium*, and especially *Mycobacterium* (see above), have been associated with disease in fish. Moreover, *Streptococcus*, another potentially pathogenic (non-corynebacterial) genus, prominently increased in relative abundance in wild summer fish and also in experimental fish given a summer-like diet. It might thus be speculated that mycolic acids, or other bacterially-derived immunogens, could provide a cue for responses limiting the outgrowth of dangerous bacteria (50) in the gut. Such responses might contribute to the re-adjustment of immunity we have observed in wild fish in summer and in fish exposed to a summer-like diet.

Crucially, even if individual corynebacterial or *Streptococcus* lineages were not in fact themselves pathogenic, the presence of conserved group-specific PAMPs could still act as a stimulus for immune re-adjustments (perhaps as a generalized host response to the likelihood of pathogen outgrowth rather than to a pathogenic challenge *per se*). For example, it has been observed that heat-killed (and thus non-pathogenic) *Gordonia* bacteria (a corynebacterial genus that varied in wild stickleback) stimulate teleost immunity when included in the diet (51). On the other hand, as we generally found a lack of direct association between immune expression and microbial composition *per se*, seasonal immune allocation may be an even more general adaptation to altered food intake: anticipating the outgrowth of dangerous bacteria under these conditions, but not necessarily relying on cues derived from specific bacteria. Further work is desirable in disentangling the role, or otherwise, of bacterial immunogens in driving the immunophenotypic responses we observed here.

Importantly, as set out in the introduction, we had predicted that if diet-driven immune effects are important determinants of microbiome composition, then the strength of the microbiome-immunophenotypic association should be greater in the gill than the gut. Through taking additional measurements in the gill we confirmed this prediction, providing strong evidence for significant immune-mediated control of the microbiome at mucosal surfaces in wild vertebrates. As set out in the Materials and methods, we can discount as very unlikely an effect whereby differences in experimental food led to differential bacterial colonization of the water column—in turn driving immune-associated gene expression. In fact, our (season-specific) experimental diets resulted in consistent immune-associated gene expression changes across tissues that were very similar to seasonal systemic gene expression changes seen in wild fish consuming corresponding diets. As seasonal bacterial colonization of the water column in the wild (oligotrophic) upland lake habitat would be very unlikely to be the same as that occurring in experiment tanks, an external water bacteria driver for the observed immune expression changes is very unlikely.

In summary, we found that season-specific diet was a very powerful driver of systemic immunophenotypic variation in wild fish. This effect occurred largely independently of the bulk OTU composition of the commensal bacterial community (which was also driven by diet) and of host condition, suggesting neither of these, in itself, was a key constraint on immune allocation under normal conditions. Nonetheless, through observations in multiple anatomical compartments, differentially exposed to the direct effects of food and immunity, we found evidence of significant diet-driven immune control of bacterial community composition in mucus layers. This points to the interactive nature of the host-microbiome relationship, and is the first time, to our knowledge, that this causal chain (diet → immunity → microbiome) has been demonstrated in wild vertebrates. We note that microbiome effects on immunity are not excluded (and are perhaps difficult to detect if microbial influences stem from phylogenetically flexible functional consortia or metabolic activities). Indeed, we identified outgrowth of potentially dangerous bacteria as a consequence of a more animal-protein-rich diet, indicating that this may be part of the ultimate explanation (if not necessarily a proximal cue) for the dramatic immune re-adjustments that we saw in response to diet change. In general, our study highlights the potential for control of individual disease risk in wild or managed animals through manipulation of the diet (52–55), but also how diet change may be intimately linked to the host's control of potentially disease-causing microbes within the normal microbiome.

## Ethics Statement

Work conformed to U.K. Home Office (HO) regulations and was approved by the animal welfare committee of the Institute of Biological, Environmental and Rural Sciences (IBERS), Aberystwyth University.

## Author Contributions

IF contributed to designing the research, conducted molecular work, analyzed the data, and contributed to writing the paper. JT carried out bioinformatic work and contributed to writing the paper. JJ applied for funding, designed and managed the research, contributed to molecular and bioinformatic work, analyzed the data, and wrote the paper.

### Conflict of Interest Statement

The authors declare that the research was conducted in the absence of any commercial or financial relationships that could be construed as a potential conflict of interest.
