# Supplementary material for: Diet in the Driving Seat: Natural Diet-Immunity-Microbiome Interactions in Wild Fish
Source: Front Immunol. 2019 Feb 19;10:243. doi: 10.3389/fimmu.2019.00243 (PMC6389695; doi:10.3389/fimmu.2019.00243)
Supplement: Supplementary file 1 [file Data_Sheet_1.docx]

**Supplementary Materials and methods, supplementary tables and supplementary figures**

**Supplementary Materials and methods**

**Additional information – bacterial reads extracted from RNAseq analysis.**

Extraction controls in our RNAseq analysis contained very low numbers of reads. Objective identification of possible contaminants in samples was achieved by applying the *R* package *decontam* (frequency method). This identifies characteristic signatures of contaminating operational taxonomic units (OTUs) through correlation with sample nucleic acids concentrations in pre-sequencing steps (here we used the kit RNA yield). The OTUs from which inference is drawn in the main text (*Tsukamurella*, *Gordonia* and *Streptococcus* OTUs) were not identified as contaminants. Table S1 below shows the numbers of bacterial reads assigned to OTUs for each sample and control.

**Additional information – bacterial reads from 16S amplicon analysis**

Pure extraction controls did not amplify at the PCR step in our 16S amplicon analysis. We analyzed 2 controls where trace amounts of sample had been passed through the DNA extraction kit and then subject to all subsequent steps (kit DNA yields 0.24-0.27 ng/μl, compared to 1.7-54.1 [mean 22.2] ng/μl for main series samples). We again applied the *decontam* package (as above) to our sample set (including the controls), basing analyses (separately) on the concentration of amplified DNA and the kit DNA yield. The OTUs from which inference is drawn in the main text (Corynebacterial and *Streptococcus* OTUs) were not identified as contaminants in either analysis. Table S3 below shows the numbers of bacterial reads assigned to OTUs for each sample.

**QPCR measurements of gene expression in spleen, gill, liver and skin**

RNA was extracted from samples previously preserved in RNA stabilization solution (RNAlater; ThermoFisher) using the RNAqueous™-Micro Total RNA Isolation Kit (ThermoFisher), following manufacturer’s instructions. The tissues (spleen, gill, liver and skin) were dissected from individual fish under a binocular microscope and manually homogenized in kit lysis buffer. RNA extracts were DNAse treated (DNA-free™ system, ThermoFisher) and converted to cDNA using the High-Capacity RNA-to-cDNA™ Kit (ThermoFisher), according to manufacturer’s instructions, including reverse transcription negative (RT-) controls for a subsample. Assays were pipetted onto 384 well plates by a robot (Pipetmax, Gilson) using a custom programme and run on a QuantStudio 6-flex Real-Time PCR System (ThermoFisher) at the machine manufacturers default real-time PCR cycling conditions. Reaction size was 10 µl, incorporating 1 µl of template and Applied Biosystems™ Fast SYBR™ Green Master Mix (ThermoFisher) and primers at the machine manufacturer’s recommended concentrations. Samples for each organ were assayed, with treatment groups dispersed evenly, over 2 plates. Each plate contained all target gene expression assays applied for the respective organ and both endogenous control gene (*yipf4*, *acvlr1*) assays, for experimental samples (in duplicate) and a calibrator sample (in triplicate). Primers used are those reported in [1]. In addition, no template controls for each gene were included on each plate. Template cDNA (see above) was diluted 1/20 prior to assay. The calibrator sample (identical on each plate) was created by pooling cDNA derived from whole fish RNA extracts from wild sticklebacks captured in summer. Relative gene expression values used in analyses are RQ values calculated by the QuantStudio 6-flex machine software according to the ∆∆Ct method, indexed to the calibrator sample. Melting curves and amplification plots were individually inspected for each well replicate to confirm specific amplification.

1. Brown M, Hablutzel P, Friberg IM, Thomason AG, Stewart A, Pachebat JA, et al. Seasonal immunoregulation in a naturally-occurring vertebrate*.* BMC Genomics. 2016;17:369.

**Table S1.** Numbers of bacterial reads identified in each RNAseq sample (only including OTUs with >5 reads).

| Sample identifier | Type | Season | Reads assigned |
| --- | --- | --- | --- |
| FG_S_1 | Sample | Summer | 4195 |
| FG_S_2 | Sample | Summer | 5879 |
| FG_S_3 | Sample | Summer | 4879 |
| FG_S_4 | Sample | Summer | 3879 |
| FG_S_5 | Sample | Summer | 5502 |
| FG_S_6 | Sample | Summer | 10685 |
| FG_S_7 | Sample | Summer | 5047 |
| FG_S_8 | Sample | Summer | 5296 |
| FG_W_1 | Sample | Winter | 5847 |
| FG_W_1 | Sample | Winter | 5205 |
| FG_W_2 | Sample | Winter | 5401 |
| FG_W_3 | Sample | Winter | 5210 |
| FG_W_4 | Sample | Winter | 4754 |
| FG_W_5 | Sample | Winter | 7377 |
| FG_W_6 | Sample | Winter | 7561 |
| FG_W_7 | Sample | Winter | 5936 |
| FG_W_8 | Sample | Winter | 5798 |
| FG_W_9 | Sample | Winter | 4476 |
| FG_W_10 | Sample | Winter | 5847 |
| RH_S_1 | Sample | Summer | 5108 |
| RH_S_2 | Sample | Summer | 5333 |
| RH_S_4 | Sample | Summer | 5497 |
| RH_S_5 | Sample | Summer | 5455 |
| RH_S_6 | Sample | Summer | 5722 |
| RH_S_7 | Sample | Summer | 6786 |
| RH_S_8 | Sample | Summer | 5546 |
| RH_S_9 | Sample | Summer | 5340 |
| RH_W_1 | Sample | Winter | 5577 |
| RH_W_2 | Sample | Winter | 7272 |
| RH_W_3 | Sample | Winter | 5234 |
| RH_W_4 | Sample | Winter | 5350 |
| RH_W_5 | Sample | Winter | 5455 |
| RH_W_6 | Sample | Winter | 9408 |
| RH_W_7 | Sample | Winter | 5360 |
| RH_W_8 | Sample | Winter | 6807 |
| RH_W_9 | Sample | Winter | 5042 |
| RH_W_10 | Sample | Winter | 5420 |
| Control1 | Control |  | 0 |
| Control2 | Control |  | 19 |
| Control3 | Control |  | 8 |

**Table S2.** Sample sizes for tissue-specific gene expression variables (QPCR measurements) in diet experiment.

| Gene | Spleen | | Gill | | Liver | | Fin | |
| --- | --- | --- | --- | --- | --- | --- | --- | --- |
| Diet treatment | S | W | S | W | S | W | S | W |
| *cd8a* | 14 | 14 | 14 | 16 | 14 | 16 | 14 | 14 |
| *foxp3b* | 14 | 14 | 14 | 16 | 14 | 16 | 14 | 12 |
| *orai1* | 14 | 14 | 14 | 16 | 14 | 16 | nm | nm |
| *tbk1* | 14 | 14 | 14 | 16 | 14 | 16 | 13 | 14 |
| *il1r*-like | 14 | 14 | 14 | 16 | 14 | 16 | 14 | 14 |

S = summer-like diet; W = winter-like diet; nm = not measured.

**Table S3.** Numbers of bacterial reads identified in each 16S amplicon sample (only including OTUs with >5 reads).

| Sample identifier | Type | Diet | Organ | Reads assigned |
| --- | --- | --- | --- | --- |
| V15G | Sample | Winter-like | Gill | 79427 |
| V10G | Sample | Winter-like | Gill | 96173 |
| V13G | Sample | Winter-like | Gill | 124608 |
| V11G | Sample | Winter-like | Gill | 65032 |
| V9G | Sample | Winter-like | Gill | 96711 |
| V16G | Sample | Winter-like | Gill | 147539 |
| V8G | Sample | Winter-like | Gill | 55184 |
| V14G | Sample | Winter-like | Gill | 70109 |
| V12G | Sample | Winter-like | Gill | 50822 |
| V8I | Sample | Winter-like | Gut | 48996 |
| V14I | Sample | Winter-like | Gut | 109025 |
| V12I | Sample | Winter-like | Gut | 39908 |
| V4I | Sample | Winter-like | Gut | 53085 |
| V3I | Sample | Winter-like | Gut | 193036 |
| V11I | Sample | Winter-like | Gut | 122487 |
| V16I | Sample | Winter-like | Gut | 114542 |
| V10I | Sample | Winter-like | Gut | 85283 |
| V1I | Sample | Winter-like | Gut | 34099 |
| V9I | Sample | Winter-like | Gut | 54391 |
| V13I | Sample | Winter-like | Gut | 216127 |
| V7I | Sample | Winter-like | Gut | 70567 |
| V2I | Sample | Winter-like | Gut | 151024 |
| V6I | Sample | Winter-like | Gut | 110211 |
| C12G | Sample | Summer-like | Gill | 73138 |
| C7G | Sample | Summer-like | Gill | 71719 |
| C13G | Sample | Summer-like | Gill | 33979 |
| C10G | Sample | Summer-like | Gill | 40348 |
| C14G | Sample | Summer-like | Gill | 5810 |
| C16G | Sample | Summer-like | Gill | 75877 |
| C11G | Sample | Summer-like | Gill | 83706 |
| C15G | Sample | Summer-like | Gill | 74663 |
| C9G | Sample | Summer-like | Gill | 72113 |
| C2I | Sample | Summer-like | Gut | 76366 |
| C13I | Sample | Summer-like | Gut | 105434 |
| C1I | Sample | Summer-like | Gut | 82455 |
| C6I | Sample | Summer-like | Gut | 87617 |
| C8I | Sample | Summer-like | Gut | 88795 |
| C9I | Sample | Summer-like | Gut | 97731 |
| C15I | Sample | Summer-like | Gut | 113101 |
| C7I | Sample | Summer-like | Gut | 110597 |
| C10I | Sample | Summer-like | Gut | 89828 |
| C11I | Sample | Summer-like | Gut | 59078 |
| C3I | Sample | Summer-like | Gut | 95533 |
| C16I | Sample | Summer-like | Gut | 117401 |
| C12I | Sample | Summer-like | Gut | 31717 |
| C5I | Sample | Summer-like | Gut | 72729 |
| Control1 | Control |  |  | 54822 |
| Control2 | Control |  |  | 137974 |

**Figure S1. Diagrammatic summary of overall study design.**

**
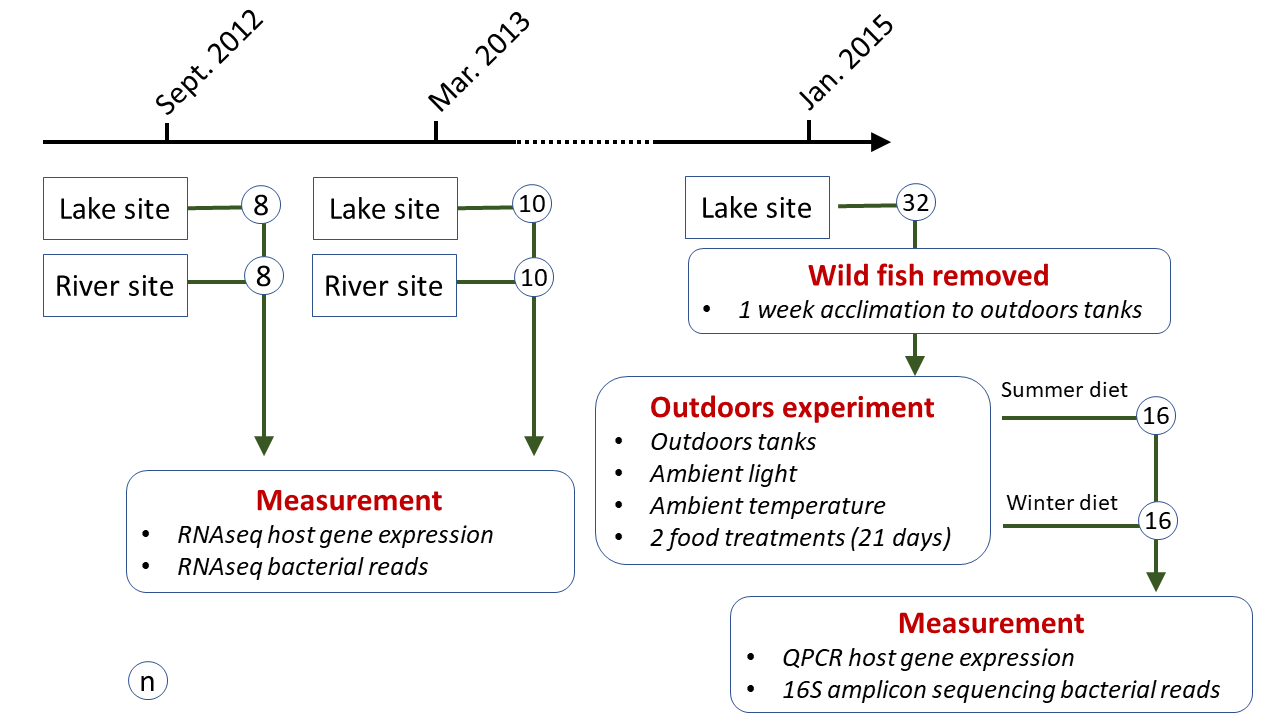
**

**Figure S2. Boxplots of raw whole-fish SR gene expression data (RNAseq measurements) from wild fish.**


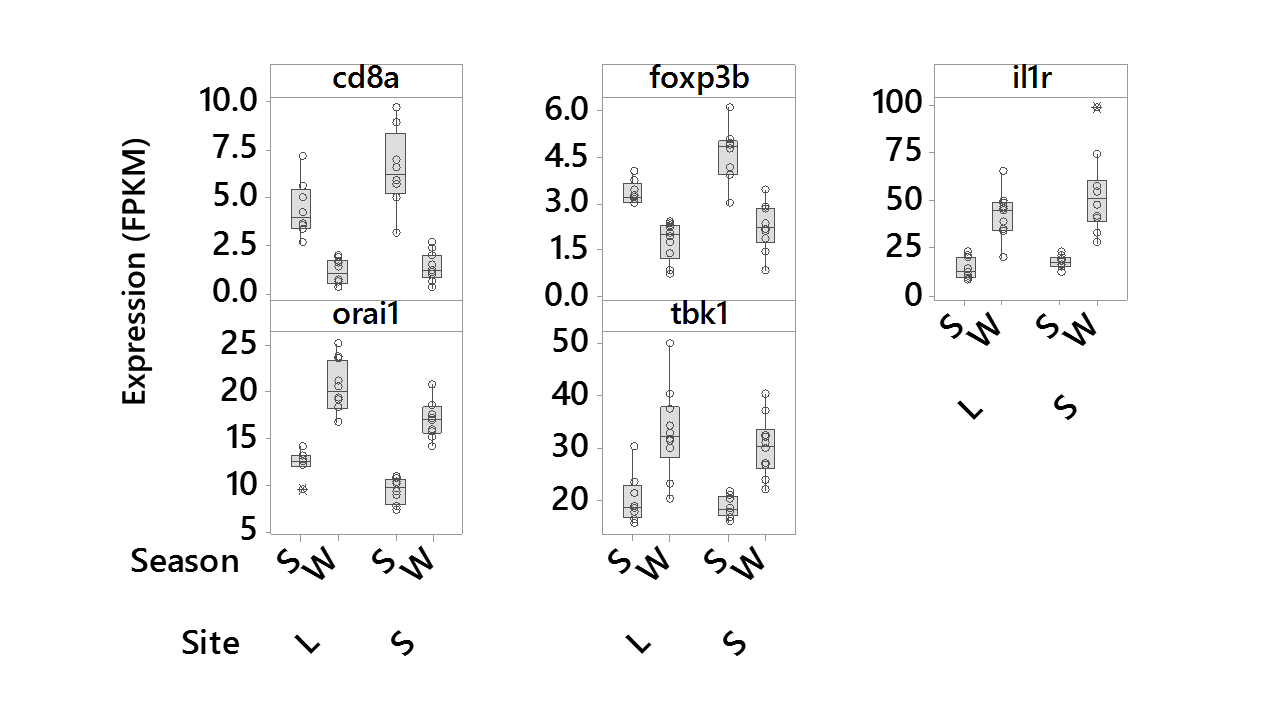
Boxplots showing observations for individual fish (circles), the sample median (line), the sample interquartile range (shaded box) and the sample range excluding outliers (whiskers). Individual outliers (points lying > 1.5 × the interquartile range outside of the 1^st^ to 3^rd^ quartile interval) are indicated as crossed points outside of the whiskers. Data plotted are raw RNAseq expression values expressed in fragments per kilobase of exon per million reads mapped (FPKM) by season (summer, S; winter, W) and site (lake, L; river, S). SR (seasonal reporter) genes were selected to represent a dominant immunome-wide seasonal trend [1].

1. Brown M, Hablutzel P, Friberg IM, Thomason AG, Stewart A, Pachebat JA, et al. Seasonal immunoregulation in a naturally-occurring vertebrate*.* BMC Genomics. 2016;17:369.


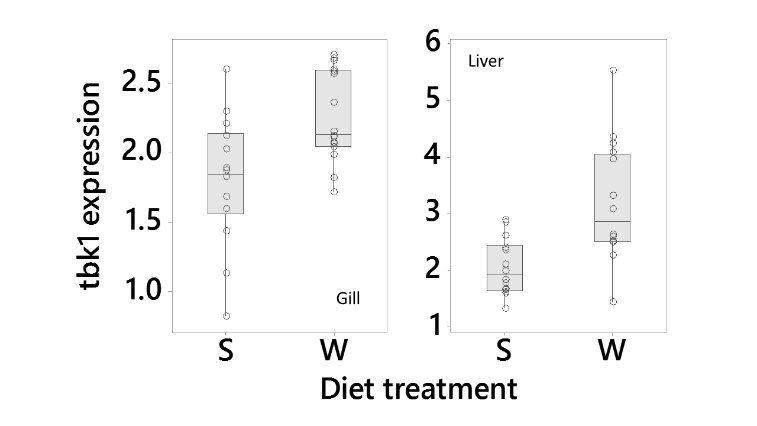

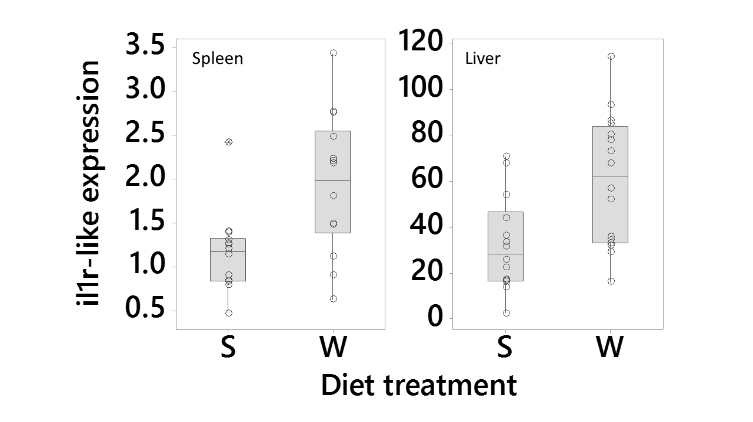

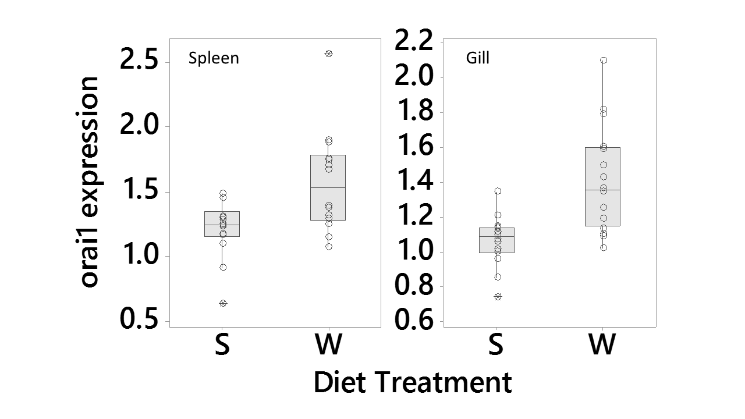

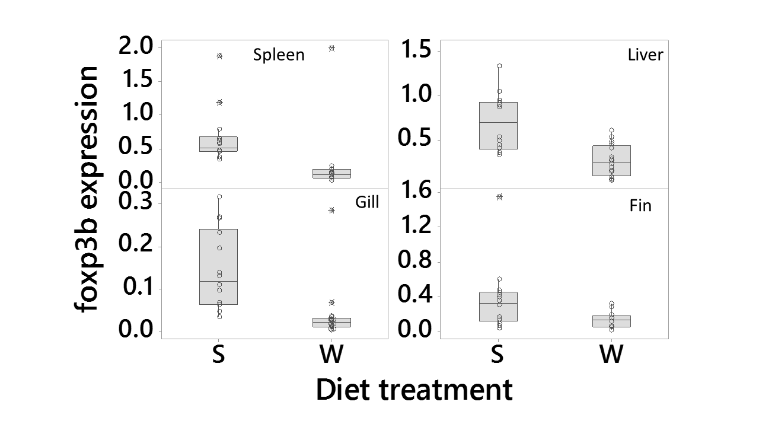

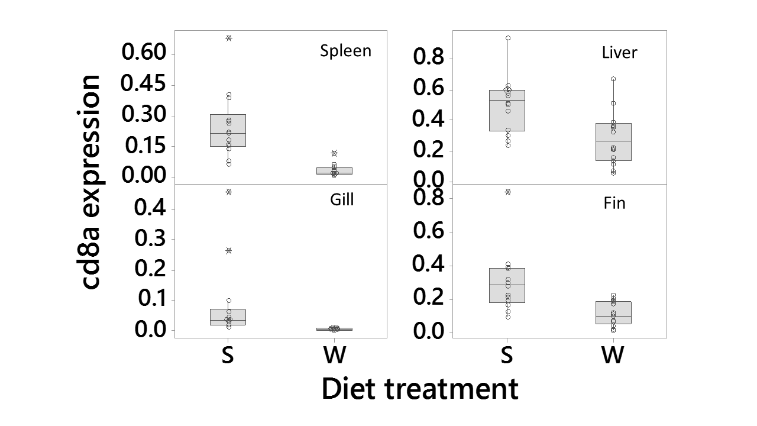
**Figure S3. Boxplots of raw tissue-specific SR gene expression data (QPCR measurements) from the diet experiment.**

Boxplots showing observations for individual fish (circles), the sample median (line), the sample interquartile range (shaded box) and the sample range excluding outliers (whiskers). Individual outliers (points lying > 1.5 × the interquartile range outside of the 1^st^ to 3^rd^ quartile interval) are indicated as crossed points outside of the whiskers. Data plotted (significant trends only, see Table 1 in main text) are raw tissue-specific relative expression values (normalised to 2 endogenous control genes and indexed to a common calibrator sample by the ∆∆Ct method) by gene (see y-axis label) and diet treatment (summer-like diet, S: winter-like diet, W). SR (seasonal reporter) genes were selected to represent a dominant immunome-wide seasonal trend [1].

1. Brown M, Hablutzel P, Friberg IM, Thomason AG, Stewart A, Pachebat JA, et al. Seasonal immunoregulation in a naturally-occurring vertebrate*.* BMC Genomics. 2016;17:369.
